# The impact of comorbidity and stage on ovarian cancer mortality: A nationwide Danish cohort study

**DOI:** 10.1186/1471-2407-8-31

**Published:** 2008-01-29

**Authors:** Mette S Tetsche, Claus Dethlefsen, Lars Pedersen, Henrik T Sorensen, Mette Norgaard

**Affiliations:** 1 Department of Clinical Epidemiology, Aarhus University Hospital, 8000 Aarhus, Denmark; 2 Department of Gynecology, Aalborg Hospital, Aarhus University Hospital, 9000 Aalborg, Denmark; 3 Department of Epidemiology, Boston University School of Public Health, Boston, MA 02118, USA; 4 Center for Cardiovascular Research, Aalborg Hospital, Aarhus University Hospital, 9000 Aalborg, Denmark

## Abstract

**Background:**

The incidence of ovarian cancer increases sharply with age, and many elderly patients have coexisting diseases. If patients with comorbidities are diagnosed with advanced stages, this would explain the poor survival observed among ovarian cancer patients with severe comorbidity. Our aims were to examine the prevalence of comorbidity according to stage of cancer at diagnosis, to estimate the impact of comorbidity on survival, and to examine whether the impact of comorbidity on survival varies by stage.

**Methods:**

From the Danish Cancer Registry we identified 5,213 patients (> 15 years old) with ovarian cancer diagnosed from 1995 to 2003. We obtained information on comorbidities from the Danish National Hospital Discharge Registry. Vital status was determined through linkage to the Civil Registration System. We estimated the prevalence of comorbidity by stage and computed absolute survival and relative mortality rate ratios (MRRs) by comorbidity level (Charlson Index score 0, 1–2, 3+), using patients with Charlson Index score 0 as the reference group. We then stratified by stage and computed the absolute survival and MRRs according to comorbidity level, using patients with Charlson score 0 and localized tumour/FIGO I as the reference group. We adjusted for age and calendar time.

**Results:**

Comorbidity was more common among patients with an advanced stage of cancer. One- and five-year survival was higher in patients without comorbidity than in patients with registered comorbidity. After adjustment for age and calendar time, one-year MRRs declined from 1.8 to 1.4 and from 2.7 to 2.0, for patients with Charlson scores 1–2 and 3+, respectively. After adjustment for stage, the MRRs further declined to 1.3 and 1.8, respectively. Five-year MRRs declined similarly after adjustment for age, calendar time, and stage. The impact of severe comorbidity on mortality varied by stage, particularly among patients with tumours with regional spread/FIGO-stages II and III.

**Conclusion:**

The presence of severe comorbidity was associated with an advanced stage of ovarian cancer. Mortality was higher among patients with comorbidities and the impact of comorbidity varied by stage.

## Background

Ovarian cancer is the leading cause of death from gynaecological cancer in western countries. It has a poor prognosis, with five-year survival rates ranging from 26% to 51% in Europe [1]. As the incidence of ovarian cancer increases sharply with age, many patients have one or more other chronic diseases, *i.e*., comorbidities [2,3]. Comorbidity is an important predictor of prognosis in patients with chronic diseases, including cancer [4,5]. Among women with ovarian cancer, the presence of comorbidity may substantially influence the diagnostic work-up, alter treatment efficacy, and affect survival.

Few studies to date have examined the impact of comorbidity on ovarian cancer survival [6-11]. The presence of comorbidity at time of diagnosis was found to have a negative impact on prognosis and survival in two studies [7,9], but not in others [6,8,10,11]. Based on hospital discharge registry data, we recently reported that one-year mortality in a Danish regional study was twice as high in ovarian cancer patients with severe comorbidity as in those without recorded comorbidity [12]. However, the study was limited by lack of information on the stage of cancer and by our inability to exclude patients with borderline tumours.

The presence of comorbidities may influence stage at diagnosis, which in turn is a strong predictor of survival in ovarian cancer patients. It is possible that patients with comorbidities experience delay in diagnosis, resulting in a more advanced cancer stage. We thus hypothesized that poorer survival among ovarian cancer patients with severe comorbidity, compared with those without comorbid conditions, may be explained by a higher prevalence of advanced cancer at diagnosis.

The Danish Cancer Registry records stage information on incident ovarian cancers. Using data from this Registry, we conducted a nationwide study to determine the prevalence of comorbidity by stage of ovarian cancer, to estimate the impact of comorbidity on survival and mortality, controlling for cancer stage, and to examine whether the effect of comorbidity on ovarian cancer mortality varies by cancer stage at diagnosis.

## Methods

We conducted this nationwide study in Denmark, which has approximately 5.4 million inhabitants, from January 1, 1995 to December 31, 2005. The entire Danish population has free access to tax-supported medical care, including hospitalization. There is practically no private inpatient ovarian cancer treatment available. In 2004, surgery for ovarian cancer took place in 52 hospital departments [13], five of which were gynecologic oncologic centers. Thus, treatment of this cancer is quite decentralized in the Danish setting.

### Identification of ovarian cancer patients

#### The Danish Cancer Registry

The Danish Cancer Registry has kept records of all patients in Denmark with malignant neoplasms since 1943 [14]. The cancer stage at the time of diagnosis is reported to the Danish Cancer Registry as either localized tumour, tumour with regional spread, tumour with distant metastases or, alternatively, by stage according to the FIGO classification [15]. For tumours categorized as regional spread, we were unable to determine whether it was a FIGO-stage II or FIGO-stage III tumour, therefore we used the scheme of Kjaerby-Thygesen *et al*. to categorize ovarian cancer cases into four groups: (a) localized tumours/FIGO-stage I tumours; (b) tumours with regional spread/FIGO-stage II and III tumours; (c) tumours with distant metastases/FIGO-stage IV tumours (also referred to as "advanced stage" below); and (d) tumours with unspecified stage [16]. The Cancer Registry provided dichotomized data on cancer treatment within the first four months following diagnosis, such as surgery (yes/no), chemotherapy (yes/no), radiation (yes/no), and other (yes/no) [17]. The Registry also contains information on histological types.

We searched the Danish Cancer Registry for patients with a first-time ovarian cancer diagnosis [International Classification of Diseases (ICD), 7^th ^revision, codes 175.0, 175.1, 175.2, 175.3, 375.0, 475.0, or 875.0] [18] between 1 January, 1995 and 31 December, 2003. We omitted children since ovarian cancer seldom occurs in childhood and often has a different clinical picture than in adults. Eight patients younger than 15 years thus were excluded from the analysis.

### Data on comorbidity

#### The Danish Hospital Discharge Registry

The Danish National Hospital Discharge Registry [19] contains information on all patients discharged from non-psychiatric hospitals in Denmark since 1977. Data on outpatient visits have been included since 1995 [20]. Information initially is collected in county-specific hospital discharge registries immediately upon discharge, and then is transferred to the National Registry. Data in this Registry, which covers more than 99% of all non-psychiatric discharges nationwide [19], are used routinely to monitor hospital admissions and discharges, waiting lists, operations, and treatment. Records include civil registration number (CPR), dates of admission and discharge, surgical procedure(s) performed, and up to 20 discharge diagnoses, which are classified by physicians according to the Danish version of the International Classification of Diseases (ICD) (8th revision until the end of 1993 and 10th revision thereafter) [19].

For each patient with ovarian cancer identified from the Danish Cancer Registry, we extracted all discharge diagnoses documented in the Hospital Discharge Registry between January 1, 1977 and the date of ovarian cancer diagnosis. We obtained 18 to 26 years of hospitalization history for each patient, depending on date of diagnosis, and used this information to compute the Charlson Comorbidity Index [4]. This Index is a weighted index of the number and the seriousness of comorbid diseases [21], which has been widely used in studies of cancer patients [5,22-25]. Because ovarian cancer defined our study cohort, we excluded this diagnosis from Index calculations. The comorbid conditions in the 5,213 ovarian cancer patients are shown in Table 1.

**Table 1:** Comorbid conditions in the 5,213 ovarian cancer patients

Conditions	Total (%)	Charlson score
Myocardial infarct	113 (2.2%)	1
Congestive heart failure	117 (2.2%)	1
Peripheral vascular disease	112 (2.2%)	1
Cerebrovascular disease	229 (4.4%)	1
Dementia	17 (0.3%)	1
Chronic pulmonary disease	232 (4.5%)	1
Connective tissue disease	137 (2.6%)	1
Ulcer disease	180 (3.5%)	1
Mild liver disease	47 (0.9%)	1
Diabetes type 1 or type 2	124 (2.4%)	1
Hemiplegia	8 (0.2%)	2
Moderate or severe renal disease	39 (0.8%)	2
Diabetes with end organ damage type 1 or type 2	50 (1.0%)	2
Any tumour (not ovarian cancer)	490 (9.4%)	2
Leukemia	9 (0.2%)	2
Lymphoma	13 (0.3%)	2
Moderate or severe liver disease	11 (0.2%)	3
Metastatic solid tumour	160 (3.1%)	6
AIDS	0	6

Patients were categorized in three groups according to their Charlson Index comorbidity score: score 0 (no recorded comorbidity), score 1–2 (moderate comorbidity), and score 3 or more (severe comorbidity).

#### Record linkage

Since 1968, a unique 10-digit civil registration number has been assigned to each Danish resident by the Central Office of Civil Registration. Use of this number permits data linkage between registries. The Civil Registration System is updated daily, and contains information on vital status, date of death, and the residence of all Danish residents [26]. We used information from this Registry to follow patients until death, emigration, or 31 December 2005, whichever came first, and to compute all-cause mortality.

### Statistical analyses

#### Stage of cancer associated with comorbidity

We constructed contingency tables of cancer stage and comorbidity level. Using Mantel-Haenszel methods [27], we then computed age-adjusted prevalence ratios to compare the prevalence of distant metastases/FIGO IV among patients with comorbidity with that among patients without comorbidity.

#### Comorbidity, stage of cancer and survival

We computed Kaplan-Meier survival curves for each Charlson comorbidity group and cancer stage (localized/FIGO-stage I tumours, tumours with regional spread/FIGO-stage II and III tumours, and tumours with distant metastases/FIGO-stage IV tumours).

Using Cox proportional hazards regression analysis, we computed one- and five-year crude and adjusted hazard ratios as a measure of mortality rate ratios [28]. Patients with no registered comorbidity served as the reference group. We adjusted first for age and calendar time (3-year calendar periods) and then for cancer stage.

Design variables were created for the 12 combinations of stage and comorbidity. For each stratum we computed one- and five-year survival using Kaplan-Meier product limit methods [29], absolute survival was defined as the proportion of the patients who were still alive one- or five-years after diagnosis. Cox proportional hazards regression analysis was used to compare mortality rates. Patients with localized tumours/FIGO-stage I tumours and no registered comorbidity served as the reference group. We adjusted for age and year of diagnosis (3-year calendar periods).

In addition, the analyses were repeated adjusting for treatment. We also conducted analyses restricted to ovarian cancer patients who had received either surgery, chemotherapy or both within the first four months after diagnosis.

We verified the assumption of proportional hazards in the Cox model graphically. Estimates are provided with their corresponding 95% Confidence Intervals (95% CI). Analyses were performed using STATA version 9.2 (Stata Corporation, College Station, Tx, USA).

## Results

### Descriptive data

We identified 5,213 patients above 15 years of age diagnosed with ovarian cancer from 1995 to 2003. Of these, 3,727 (72%) had no comorbidity recorded in the National Hospital Discharge Registry, 1,116 (21%) had Charlson score 1–2, and 370 (7%) had Charlson score 3+ (Table 2). The median age was 62 years in patients without comorbidity, 70 years in patients with Charlson score 1–2, and 71 years in patients with Charlson score 3+. Patients without comorbidity were more frequently treated with surgery/chemotherapy (47%) than patients with severe comorbidity (27%) (Table 2).

**Table 2:** Characteristics of ovarian cancer patients, N = 5,213.

	**Charlson Comorbidity score**		
	**Comorbidity 0**	**Comorbidity 1–2**	**Comorbidity 3+**

**Number**	3,727 (72%)	1,116 (21%)	370 (7%)
**Median age**, years	62	70	71
(25% – 75% percentile)	(52–72)	(59–77)	(62–78)
**Age groups**			
< 50 years	726 (19%)	96 (9%)	21 (6%)
50–69 years	1,897 (51%)	474 (42%)	153 (41%)
70–89 years	1,079 (29%)	521 (47%)	188 (51%)
90+ years	25 (< 1%)	25 (2%)	8 (2%)
**Year of diagnosis**			
1995–1997	1,278 (74%)	336 (19%)	119 (7%)
1998–2000	1,300 (71%)	406 (22%)	119 (7%)
2001–2003	1,149 (69%)	374 (23%)	132 (8%)
**Extent of cancer**			
Localized/FIGO I	774 (21%)	184 (16%)	37 (10%)
Regional/FIGO II and III	1,757 (47%)	492 (44%)	157 (42%)
Distant metastases/FIGO IV	1,002 (27%)	338 (30%)	140 (38%)
Unspecified/missing	194 (5%)	102 (9%)	36 (10%)
**Histological type**			
Epithelial	3,422 (92%)	985 (88%)	322 (87%)
Non-epithelial	305 (8%)	131 (12%)	48 (13%)
**Treatment**			
Surgery/Chemotherapy	1,753 (47%)	378 (34%)	101 (27%)
Surgery	1,416 (38%)	442 (40%)	132 (36%)
Chemotherapy	230 (6%)	94 (8%)	32 (9%)
Other*	12 (< 1%)	9 (< 1%)	6 (2%)
No/Symptomatic	285 (8%)	178 (16%)	94 (25%)
Missing	31 (< 1%)	15 (1%)	5 (1%)

*Hormonal treatment, radiation, other not specified

An association between comorbidity and advanced stage, for whom information on stage of disease was available, was found only among patients with severe comorbidity. The age-adjusted prevalence ratio was 1.0 (95% CI, 0.9–1.1) among patients with moderate comorbidity. Among patients with severe comorbidity, 42% had distant metastases/FIGO IV, compared to 28% of patients without comorbidity (age-adjusted prevalence ratio = 1.3, 95% CI, 1.1–1.5).

### Comorbidity survival and mortality

Estimates of one- and five-year survival and MRR by level of comorbidity are shown in Table 3. One- and five-year survival was higher in patients without comorbidity than in patients with registered comorbidity. After adjustment for age and calendar time, one-year MRRs declined from 1.8 to 1.4 for patients with Charlson score 1–2 and from 2.7 to 2.0 for patients with Charlson score 3+. When adjustment for stage was added to the model, the MRRs further declined to 1.3 for patients with Charlson score 1–2 and 1.8 for patients with Charlson score 3+. Five-year MRRs declined similarly after adjusting for age, calendar time, and stage.

**Table 3:** One- and five-year mortality rate ratios (MRRs) by level of comorbidity. The corresponding 95% confidence interval is given in parentheses.

	**Charlson Comorbidity score**		
	**0**	**1–2**	**3+**

N (%)	3,727 (72%)	1,116 (21%)	370 (7%)
1-year			
Survival in %	73 (71–74)	58 (55–60)	44 (39–49)
MRR	1 (ref.)	1.8 (1.6–2.0)	2.7 (2.3–3.1)
Adj. MRR*	1 (ref.)	1.4 (1.2–1.5)	2.0 (1.7–2.3)
Adj. MRR**	1 (ref.)	1.3 (1.2–1.5)	1.8 (1.6–2.1)
5-year			
Survival in %	37 (36–39)	24 (21–27)	12 (8–16)
MRR	1 (ref.)	1.5 (1.4–1.6)	2.3 (2.1–2.6)
Adj. MRR*	1 (ref.)	1.3 (1.2–1.4)	1.8 (1.6–2.1)
Adj. MRR**	1 (ref.)	1.2 (1.1–1.4)	1.7 (1.5–1.9)

*Adjusted for age and calendar time.

**Adjusted for age, calendar time and stage.

### Comorbidity, stage of cancer and survival

**Figure 1:**
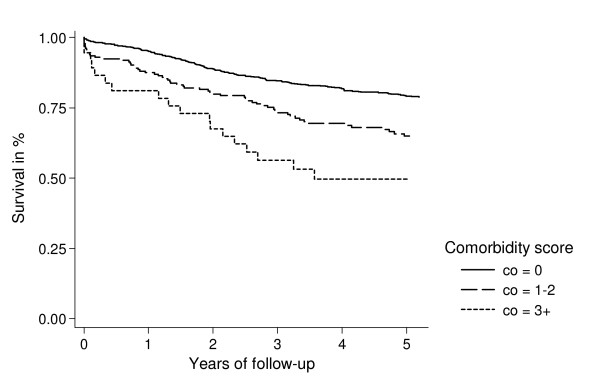
Kaplan-Meier survival curves for ovarian cancer patients with localized tumour/FIGO stage I, according to presence of comorbidity at time of diagnosis.

**Figure 2:**
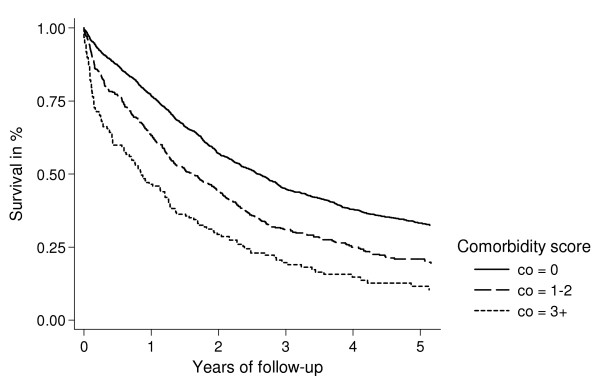
Kaplan-Meier survival curves for ovarian cancer patients with regional spread/FIGO stage II and III, according to presence of comorbidity at time of diagnosis.

**Figure 3:**
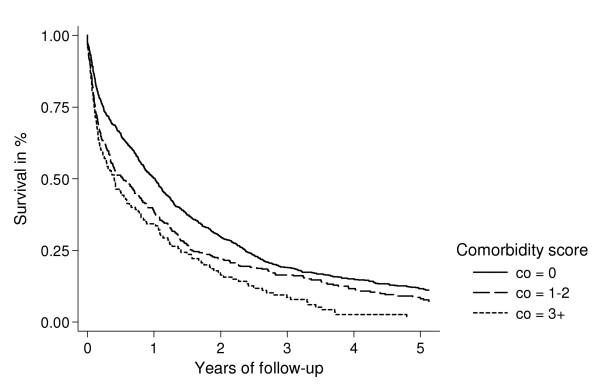
Kaplan-Meier survival curves for ovarian cancer patients with distant metastases/FIGO stage IV, according to presence of comorbidity at time of diagnosis.

Figures 1, 2, 3 show survival curves for patients with ovarian cancer by level of comorbidity at time of diagnosis. For all stages, survival was higher in patients without comorbidity than in patients with comorbidity. One- and five-year survivals are shown in Tables 4 and 5.

**Table 4:** One-year survival and one-year mortality rate ratio (MRR) by level of comorbidity according to the stage of cancer and adjusted for age and calendar time.

	**Charlson Comorbidity score**		
	0	1–2	3+

**Localized tumour/FIGO-stage I**			
Number	774	184	37
Median age, years	56	67	72
Survival in %	95 (93–97)	88 (82–92)	81 (64–91)
Crude MRR	1 (ref.)	2.8 (1.6–4.6)	4.5 (2.0–10.0)
Adj. MRR	1 (ref.)	2.1 (1.2–3.5)	2.7 (1.2–6.2)
**Regional spread/FIGO-stage II, III**			
Number	1,757	492	157
Median age, years	62	68	69
Survival in %	77 (75–79)	63 (59–67)	47 (39–54)
Crude MRR	5.3 (3.8–7.4)	9.2 (6.5–13.2)	16.0 (10.9–23.6)
Adj. MRR	4.8 (3.5–6.8)	7.1 (5.0–10.1)	12.3 (8.3–18.1)
**Distant metastases/FIGO-stage IV**			
Number	1,002	338	140
Median age, years	66	71	71
Survival in %	50 (47–53)	38 (33–44)	34 (27–42)
Crude MRR	14.1 (10.1–19.7)	20.4 (14.4–28.9)	23.4 (16.0–34.2)
Adj. MRR	11.6 (8.3–16.2)	13.9 (9.8–19.8)	15.7 (10.7–23.1)
**Unspecified**			
Number	194	102	36
Median age, years	66	75	71
Survival in %	62 (55–68)	40 (31–50)	33 (19–49)
Crude MRR	10.1 (6.8–15.0)	19.2 (12.8–28.9)	25.0 (15.0–41.8)
Adj. MRR	8.1 (5.5–12.1)	11.2 (7.4–16.8)	15.3 (9.1–25.7)

The reference group was patients with localized tumour/FIGO I and no registered comorbidity. The corresponding 95% confidence interval is given in parentheses.

**Table 5:** Five-year survival and five-year all-cause mortality rate ratio (MRR) by level of comorbidity according to the FIGO-stages and adjusted for age and calendar time.

	**Charlson Comorbidity score**		
	0	1–2	3+

**Localized tumour/FIGO-stage I**			
Survival in %	79 (76–82)	65 (57–72)	50 (32–65)
Crude MRR	1 (ref.)	1.8 (1.4–2.5)	3.2 (2.0–5.3)
Adj. MRR	1 (ref.)	1.6 (1.2–2.1)	2.3 (1.4–3.8)
**Regional spread/FIGO-stage II, III**			
Survival in %	33 (31–36)	21 (17–25)	12 (7–18)
Crude MRR	4.5 (3.8–5.4)	6.7 (5.6–8.1)	10.1 (8.0–12.7)
Adj. MRR	4.4 (3.7–5.2)	5.7 (4.7–6.9)	8.6 (6.8–10.9)
**Distant metastases/FIGO-stage IV**			
Survival in %	12 (10–14)	8 (5–12)	2 (0.4–6)
Crude MRR	9.7 (8.1–11.5)	12.4 (10.2–15.0)	15.9 (12.6–20.0)
Adj. MRR	8.9 (7.5–10.6)	9.7 (8.0–11.8)	12.1 (9.6–15.4)
**Unspecified**			
Survival in %	36 (29–43)	17 (10–26)	14 (5–29)
Crude MRR	5.1 (4.0–6.5)	9.8 (7.5–12.8)	11.8 (8.0–17.5)
Adj. MRR	4.6 (3.6–5.8)	6.8 (5.2–8.9)	8.0 (5.4–12.0)

The reference group was patients with localized tumour/FIGO I and no registered comorbidity. The corresponding 95% confidence interval is given in parentheses.

For all stages, we found higher crude MRRs among patients with comorbidity compared with patients without comorbidity. While MRRs declined after adjustment for age and calendar time, they remained higher among patients with comorbidity (Tables 4 and 5).

We found a varying effect of severe comorbidity on one-year mortality according to stage of ovarian cancer. Tumours with regional spread/FIGO stage II and III further increased the impact of severe comorbidity on mortality (Table 4). Compared with patients with no registered comorbidity and localized tumour/FIGO I, patients with severe comorbidity and this cancer stage had an adjusted MRR of 2.7, patients with no registered comorbidity and tumours with regional spread/FIGO stage II and III had an adjusted MRR of 4.8, and patients with severe comorbidity and tumours with regional spread/FIGO stage II and III had an adjusted MRR of 12.3. Although not as pronounced, a similar variation in the effect of severe comorbidity by stage was seen in five-year mortality (Table 5). The impact of moderate comorbidity on one- and five-year mortality varied only slightly by stage.

Including treatment in the analysis did not remove the association between severe comorbidity and mortality (data not shown), except for tumours with distant metastases/FIGO-stage IV.

Analyses restricted to patients who received either surgery, chemotherapy or both yielded results similar to analyses including all ovarian cancer patients (data not shown).

## Discussion

In this population-based nationwide study, we found a higher prevalence of comorbidity in patients with an advanced stage compared with a less advanced stage of ovarian cancer. One- and five-year mortality were nearly twice as high in patients with severe comorbidity compared to those without registered comorbidity, even after adjustment for stage. Thus the increased prevalence of more advanced stage did not entirely explain the association between greater comorbidity and higher mortality. We found further that the impact of severe comorbidity on one- and five-year mortality varied by cancer stage.

Among the strengths of our study were its large size and Denmark's uniformly organized health care system, allowing a population-based design with virtually complete follow-up. However, the accuracy of our findings depends on the quality of cancer registry and hospital discharge data. The Danish Cancer Registry is known to be more than 95% complete [14], and in a previous study in which pathology records were used to confirm diagnoses, we found that ovarian cancer diagnoses were correct in 97% of the cases in the Registry, [30]. This minimal selection bias should not be related to the presence of comorbidity, however, since comorbidity was independently recorded before the cancer diagnosis.

Cancer staging is based on a combination of pathologic, operative, and clinical assessments available at the time of diagnosis. Some misclassification of stage data may occur, which could result in residual confounding. There may also have been some inaccuracy in treatment data used in the study. These data, obtained from the Danish Cancer Registry, are limited to treatment given within the first four months following diagnosis; since Registry reporting forms are often completed in the early period of treatment planning, they may not reflect actual treatment. If complications stemming from comorbidity lead to changes in treatment, this could result in residual confounding.

We used the validated Charlson Comorbidity Index as a measure of comorbidity. When applied to administrative data information on comorbidity is based on ICD-codes. The comorbid diseases may be coded with different accuracy in the different administrative registries and misclassifications occur in most registries [31]. The Charlson index has been shown to have a high specificity [4], but a more variable sensitivity when compared with diagnoses abstracted from the medical charts [32]. It is thus possible that some patients with comorbid conditions may have been classified erroneously as having Charlson score 0. Similarly, patients with severe comorbidity may have been classified erroneously as having Charlson score 1–2. However, because comorbidity was independently recorded before the cancer diagnosis, any misclassification of comorbid conditions was probably unrelated to ovarian cancer stage.

Patients with comorbidities may experience a delay in diagnosis or they may actually be diagnosed earlier because they have a close relationship with the health care system. We found that the presence of severe comorbidity was associated with an advanced stage of ovarian cancer. If ovarian cancer progresses from Figo-stage I to IV this could suggest a delay in diagnosis. It has, however, been suggested that stage I and stage III may be different forms of the disease [33].

Our findings disagree with a Dutch population-based study by Mass *et al*. [6], which was restricted to approximately 500 patients with FIGO-stage II and III ovarian cancer. Using a slightly modified Charlson Comorbidity Index and adjusting for treatment, age, stage, and period of diagnosis, they concluded that comorbidity did not influence prognosis. Other cohort studies have had similar findings [8,10,11]. A Norwegian population-based cohort study (N = 571) examining the impact of several possible prognostic factors on survival found that comorbidity was a prognostic factor in univariate but not multivariate analyses [11]. One reason this study did not find an association may be its adjustment for residual tumour. Presence of a residual tumour is related to the aggressiveness of surgery and if comorbidity results in less aggressive surgery, residual tumour may be an intermediate in the causal pathway from comorbidity to death. In this situation, adjustment for residual tumour would be inappropriate. The effect of comorbidity on mortality may be mediated to a large degree by higher volume of residual tumour. A Dutch population-based study (N = 1,116) that adjusted for age, stage and treatment also did not find an independent effect of comorbidity on prognosis [10]. Similarly, an American hospital-based study reported an age-, stage- and symptom stage-adjusted mortality rate ratio of 1.04 in ovarian cancer patients with comorbidity compared to those with no comorbidity [8]. Its study population consisted of 137 ovarian cancer patients recruited during a period of almost 6 years, which could have introduced selection bias [8].

In accordance with our study, a negative impact of comorbidity on ovarian cancer mortality was found in an American population-based and in a German cohort study [7,9]. In these studies the mortality rate ratios were adjusted for stage, but the impact of stage on survival was not reported. The current study corroborates the findings in our recent study [12] on this topic. Our observation of the impact of comorbidity on mortality also confirm and extend findings for other groups of cancer patients, including breast cancer, prostate cancer, colon cancer, and lung cancer patients [34].

Since stage did not entirely account for the differences in mortality in our study, other factors may explain the role of comorbidity as a negative prognostic factor. The presence of comorbidity in a cancer patient may influence treatment choices, which in turn affect prognosis and survival [10,35]. The optimal treatment of patients with ovarian cancer is surgery and chemotherapy, with regimens depending on stage [36]. In advanced stages it is important to optimally debulk the tumour, and while this extensive surgery can be performed safely in patients with comorbid conditions [3], not all such patients receive this treatment [6]. It also is possible that following established treatment guidelines is not the best strategy for patients with multiple comorbidities [37], either because they cannot tolerate the adjuvant chemotherapy necessary after surgery or because the drugs used to treat their comorbid diseases may interact with those in chemotherapy regimens. As well, the toxicity of chemotherapy may be exacerbated by the side effects of the drugs that are used to treat comorbidities [38]. While we were able to adjust for treatment using data from the Danish Cancer Registry, information was lacking on the aggressiveness of the surgery performed or use of modified chemotherapy regimens. Adjustment for treatment in the analyses did not diminish the impact of severe comorbidity on the one-year MRR. However, despite adjustment for treatment, aggressiveness of treatment could vary in the comorbidity groups, as indicated a Norwegian study [11].

Because our study addressed all-cause rather than cause-specific mortality, patients could have died from their comorbidity or other causes not related to ovarian cancer. This may also explain some of the higher mortality in patients with comorbidity. However, it is difficult to distinguish between the contributions to mortality from the ovarian cancer itself and that from cancer complications or comorbidities. An example is death from heart-disease vs. death due to chemotherapy-related aggravation of pre-existing cardiac problems.

There is a need for clinicians to be aware of the possible presence of comorbidity in ovarian cancer patients in order to improve their treatment. Patients with regional spread/FIGO stage II and III ovarian cancer are a particularly important subgroup in this context.

## Conclusion

In this population-based study of ovarian cancer patients, we found that the presence of severe comorbidity (Charlson score 3+) was associated with an advanced stage of cancer. Mortality was also higher among patients with comorbidities, but could not be entirely explained by the cancer stage.

## Competing interests

The author(s) declare that they have no competing interests.

## Authors' contributions

MST participated in the design of the study, analysis and interpretation of results and has been drafting the manuscript. CD participated in the design of the study, analysis and interpretation of data. LP participated in the analysis and interpretation of data. HTS participated in the design of the study, interpretation of results and revised the manuscript critically. MN participated in the design of the study, in the interpretation of results and revised the manuscript critically. All authors read and approved the final manuscript.

## Pre-publication history

The pre-publication history for this paper can be accessed here:


